# Hemodialysis under fire: A cross-sectional study of health and socioeconomic impacts on internally displaced patients during the 2023 Sudan conflict

**DOI:** 10.1097/MD.0000000000047152

**Published:** 2026-01-09

**Authors:** Malaz Taha, Gaffar Hussein, Shahd Jaweesh, Osama Abbadi, Faris Abdon

**Affiliations:** a Department of Community Medicine, Alfajr College for Sciences and Technology, Khartoum, Sudan; b Department of Biochemistry, Faculty of Medicine, National University, Khartoum, Sudan; c Department of Medical Biochemistry, Orotta College of Medicine and Health Sciences, Asmara, Eritrea.

**Keywords:** conflict health, dialysis access, ESRD, hemodialysis. internally displaced persons, humanitarian crisis, Sudan

## Abstract

End-stage renal disease patients rely on regular hemodialysis. The 2023 Sudan conflict displaced large numbers of patients and disrupted health services. We examined dialysis access, complications, and socioeconomic effects among internally displaced patients in Port Sudan. We conducted a descriptive cross-sectional study of 133 end-stage renal disease patients receiving hemodialysis at Port Sudan Dialysis Center. Trained interviewers collected data on demographics, dialysis frequency before vs during the conflict, missed sessions and barriers, self-reported complications, and employment/finances. Data were analyzed using descriptive statistics and chi-square tests. Participants were 58.7% male; 54.1% were aged 40 to 64 years. A decrease was observed in patients receiving 3 sessions/week (from 17.3 to 4.5%), while an increase was observed in those receiving 1 session/week (from 4.5 to 12.8%; *P* = .003). More than 70% cited transport costs/availability as major barriers, and over 85% missed at least 1 session during active fighting. Unemployment rose from 18.8% to 71.4% (*P* < .001). Overall, 73.7% reported clinical complications during the conflict period. The conflict was associated with reduced dialysis access and worsening health and economic conditions. Secured transport, protected continuity of care, and targeted financial support are immediate priorities for displaced dialysis patients.

## 1. Introduction

On April 15, 2023, armed conflict escalated in Sudan, triggering a nationwide humanitarian emergency. During the study period, Port Sudan remained under the administration of the Sudanese Armed Forces and functioned as a receiving hub for civilians displaced from Rapid Support Forces-affected areas. Health facilities, supply chains, and transport networks were widely disrupted, limiting access to essential services, including medicines and dialysis consumables.^[1,2]^ By early 2025, about 30.4 million people required assistance, and more than 10.7 million were internally displaced.^[3]^ Reports also describe extensive damage to hospitals and repeated attacks on health facilities, further reducing capacity and access.^[4,5]^ Although service disruptions have been widely reported, few studies provide patient-level data on how the war has changed dialysis frequency, missed care, clinical complications, and the economic burden among displaced hemodialysis (HD) patients.

Chronic conditions such as end-stage renal disease (ESRD) are particularly vulnerable to system shocks because patients depend on regular, time-intensive HD. Before the war, Sudan had roughly 8000 ESRD patients receiving care at more than 20 centers.^[6]^ Since the conflict began, many centers have been destroyed or operate below capacity due to power, water, and consumable shortages, as well as staff shortages, making routine thrice-weekly schedules difficult to maintain.^[5]^ Interruptions in dialysis increase the risks of hyperkalemia, fluid overload, and cardiovascular events, and displaced patients face additional barriers to reaching functioning units.^[5,6]^ Economic strain further limits access, as rising out-of-pocket costs and income losses among affected households compound the problem.^[7]^

Despite the scale of the crisis, patient-level evidence on dialysis-dependent internally displaced persons remains limited.^[6]^ This study addresses that gap by describing the change in dialysis frequency, missed sessions, barriers, self-reported clinical complications, and economic impacts among displaced ESRD patients relocating to and dialyzing in Port Sudan (Sudanese Armed Forces-controlled). Our goal is to provide evidence to inform practical responses to protect dialysis access and improve patient outcomes in conflict settings. We hypothesized that, among internally displaced ESRD patients dialyzing in Port Sudan, the war period would be associated with fewer delivered dialysis sessions per week, higher self-reported clinical complications, and worsening employment and household finances.

## 2. Materials and methods

### 2.1. Study design and setting

We conducted a descriptive, cross-sectional study to assess how the 2023 Sudan conflict affected access to HD and related health and socioeconomic outcomes among internally displaced adults with ESRD receiving maintenance dialysis at the Port Sudan Dialysis Center (Red Sea State, Sudan). Data were collected between November 2024 and April 2025.

### 2.2. Participants and eligibility criteria

**Figure 1.**
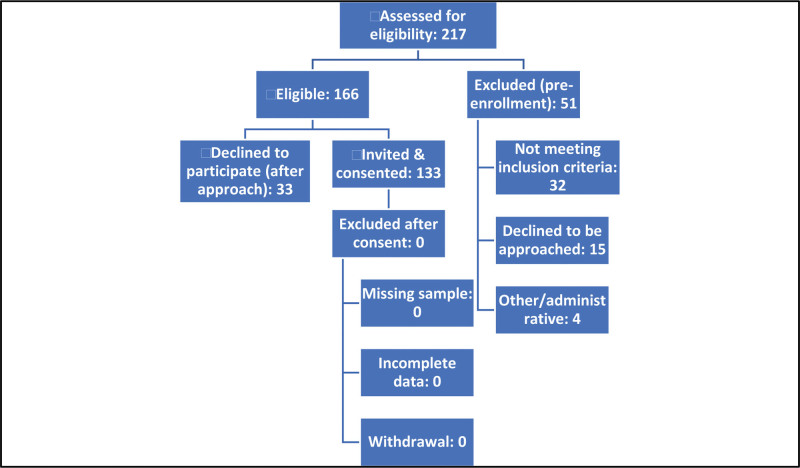
STROBE participant flow for screening, eligibility, consent, and inclusion. Screening and enrollment of internally displaced ESRD patients at Port Sudan Dialysis Center: assessed (N = 217); excluded before approach (n = 51: not meeting inclusion criteria n = 32, declined approach n = 15, other/administrative n = 4); eligible and invited (n = 166); declined after approach (n = 33); provided informed consent (n = 133); post-consent exclusions (n = 0); included in analysis (n = 133). Participation rate among approached eligible patients: 133/ (133 + 33) = 80.1%.

All adults (≥18 years) with ESRD on maintenance HD who were internally displaced due to the conflict and dialyzing at the Center during the study window were eligible. Inclusion required receipt of HD both before and during the conflict and the ability to provide informed consent. Patients who were not displaced, were not on maintenance HD, or declined to participate were excluded. A participant flow diagram summarizes screening, eligibility, consent, and analysis counts (Fig. 1).

### 2.3. Questionnaire design and data collection

We adapted items from dialysis-access and humanitarian health surveys to the local context, with input from a nephrologist and a public health specialist. The instrument underwent content review and face validity testing in a 10-patient pilot (not included in analysis); wording was refined accordingly. Trained interviewers administered the questionnaire face-to-face using a standardized script and case-report form.

### 2.4. Interviewer training and field procedures

Two community-medicine graduates served as interviewers. They completed a 1-day training covering protocol adherence, privacy, uniform question phrasing, and neutral probing. Training incorporated role-play exercises and a standardized script. A supervisor observed initial interviews to ensure fidelity; corrective feedback was provided as needed. Interviews were conducted face-to-face in a private area of the dialysis unit, typically before or after treatment.

### 2.5. Variables and definitions

Primary access outcome: number of dialysis sessions per week before the conflict (retrospective self-report) and during the conflict (current routine), categorized as 1, 2, or 3 sessions per week.

Secondary outcomes: missed sessions (any vs none during active fighting) and self-reported reasons (transport, cost, security, supply), clinical complications compatible with prolonged interdialytic intervals (e.g., fluid overload, dyspnea, hypertension, infectious events), and socioeconomic measures (employment status, household income sources, displacement-related costs, and crowding/housing stability). Demographic covariates included age, sex, and displacement origin.

### 2.6. Study size (sample size calculation)

We aimed to enroll all eligible patients during the study window. Using a conservative prevalence of 50% and 5% absolute precision at a 95% confidence level, the uncorrected sample size is $n_{0}=384$. Applying the finite-population correction with the center’s active HD roster (N = 190; census conducted during the study window) yields a minimum required sample of


$$\begin{array}{l} {}^{n}FPC=\left(n0\times N\right)/\left(n0+N-1\right)\approx 127 \end{array}$$

We enrolled 133 participants, exceeding this target.

### 2.7. Data collection and management

After consent, interviewers administered the questionnaire in Arabic. Responses were recorded on paper forms and entered into a password-protected database with range and logic checks. De-identified analytic files were prepared for analysis. Dialysis frequency during the conflict was cross-checked against available unit schedules.

### 2.8. Statistical analysis

We summarized categorical variables using counts and percentages, and continuous variables (where applicable) using means and standard deviations. Pre/post differences in dialysis frequency (3 vs 2 vs 1 sessions per week) and in employment were compared using chi-square tests. Two-sided *P* < .05 was considered statistically significant. Analyses were performed in Statistical Package for the Social Sciences (version 30).

### 2.9. Ethical considerations

The study was observational and non-interventional. Ethical approval was obtained from the Port Sudan Teaching Hospital Ethics Committee (Port Sudan, Sudan) on July 12, 2024. The committee does not issue reference numbers for descriptive cross-sectional studies; a signed approval letter is available upon request. In accordance with the approval, verbal informed consent was obtained from all participants and documented in the study log (witnessed by a nurse). Participation was voluntary, no incentives were provided, and confidentiality was maintained by de-identifying all records. The study complied with the Declaration of Helsinki and local regulations.

## 3. Results

### 3.1. Participant flow and baseline characteristics

During screening, 217 patients were assessed; 51 were excluded before approach, 166 were eligible and invited, 33 declined after approach, and 133 consented and were included in the analysis (participation rate 80.1%). The flow of participants is shown in Figure 1 (participant flowchart).

At baseline (Table 1), 58.7% were male, and just over half were aged 40 to 64 years. Most participants were married and had at least a secondary education. Slightly more than half reported at least 1 chronic condition, most commonly hypertension. All reported displacements due to the conflict. Complete distributions are provided in Table 1.

**Table 1:** Sociodemographic profile of participants (n = 133).

Variable	n (%)
Gender (%)	
Males	78 (58.7)
Female	55 (41.3)
Age Group (%)	
18–40 years	42 (31.6)
40–64 years	72 (54.1)
> 64 years	19 (14.2)
Education (%)	
Illiterate	20 (15.0)
Primary	24 (18.0)
Secondary	42 (31.6)
University	39 (29.3)
Postgraduate	8 (6.0)
Marital status (%)	
Single	27 (20.3)
Married	83 (62.4)
Divorced	13 (9.8)
Widowed	10 (7.5)
Chronic diseases (%)	
None	62 (46.6)
Diabetes	15 (11.3)
Hypertension	44 (33.1)
Diabetes and hypertension	12 (9.0)

### 3.2. Dialysis schedule and socioeconomic conditions (pre-conflict vs conflict period)

Dialysis dose shifted toward fewer weekly sessions during the conflict period: the proportion receiving 1 session per week increased, while the proportion receiving 3 sessions per week decreased (*P* = .003). Employment conditions worsened markedly, with unemployment rising and full-time employment contracting (*P* < .001). Household income sources shifted away from wages toward family support and humanitarian aid (*P* < .001). Monthly expenses > 500,000 SDG became more common (*P* = .003), and 1/3 were living in refugee centers during the conflict period (*P* < .001). Exact proportions with 95% confidence intervals are reported in Table 2.

**Table 2:** Dialysis and socioeconomic changes before and during the conflict (n = 133).

Variable	Pre‑conflict n (% [95% CI])	Conflict period n (% [95% CI])	*P* value
Dialysis sessions/week (%)			**.003**
1	6 (4.5% [2.1–9.5])	17 (12.8% [8.1–19.5])	
2	104 (78.2% [70.4–84.4])	110 (82.7% [75.4–88.2])	
3	23 (17.3% [11.8–24.6])	6 (4.5% [2.1–9.5])	
Employment status (%)			**<.001**
Unemployed	25 (18.8% [13.1–26.3])	95 (71.4% [63.2–78.4])	
Employed	47 (35.3% [27.7–43.8])	3 (2.3% [0.8–6.4])	
Free Worker/Student/Retired	61 (45.9% [37.6–54.3])	35 (26.3% [19.6–34.4])	
Main income source (%)			**<.001**
Employment	93 (69.9% [61.7–77.1])	13 (9.8% [5.8–16.0])	
Family support	39 (29.3% [22.3–37.6])	71 (53.4% [44.9–61.6])	
Humanitarian aid	1 (0.8% [0.1–4.1])	49 (36.8% [29.1–45.3])	
Monthly expenses (>500k SDG) (%)	9 (6.8% [3.6–12.4])	28 (21.1% [15.0–28.7])	**.003**
Residential status			**<.001**
Refugee Center	0 (0.0% [0.0–2.8])	44 (33.1% [25.7–41.5])	

SDG = Sudanese Pound. 95% confidence intervals (CIs) for proportions are Wilson score intervals. Percentages and CIs are calculated with n = 133 for each period.

### 3.3. Complications following disrupted dialysis

**Figure 2.**
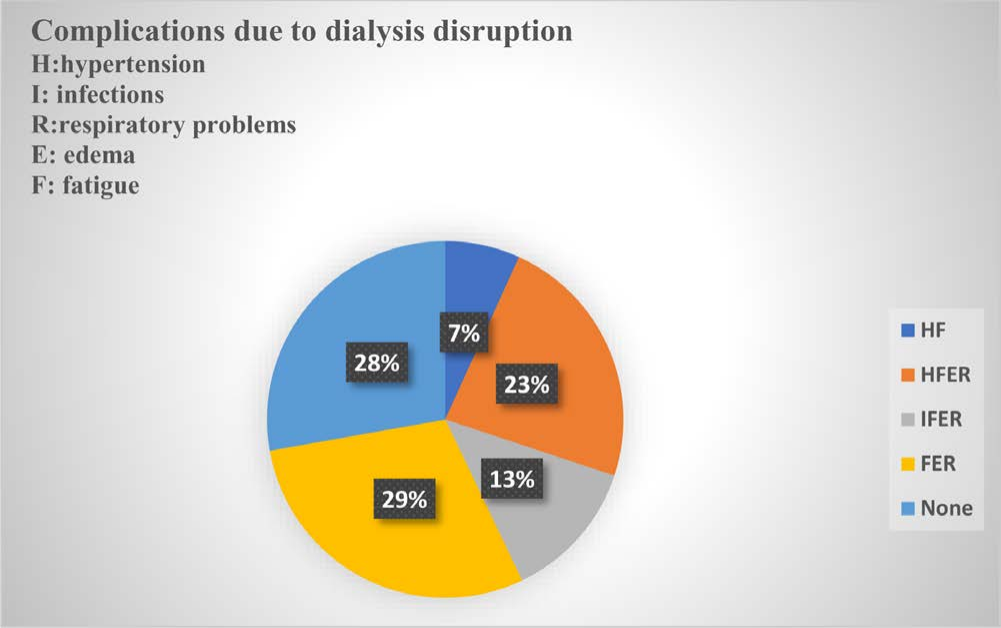
Self‑reported complications after missed dialysis sessions among internally displaced ESRD patients in Port Sudan (n = 133). This pie chart illustrates the frequency of self-reported health complications experienced by internally displaced patients with end-stage renal disease (ESRD) following missed or irregular dialysis sessions during the 2023 Sudan conflict. Patients commonly reported multiple complications (including fatigue, hypertension, swelling, shortness of breath, and infections). Only 27.8% of respondents reported no complications. The data reflect the cumulative burden of interrupted dialysis care in conflict settings.

Self-reported complications consistent with reduced dialysis dose (e.g., fluid overload/edema, dyspnea, hypertension, fatigue, infectious events) were common (Fig. 2). The distribution of reported complications is shown in the figure.

## 4. Discussion

The findings from this study show how the 2023 Sudan conflict harmed both the health and livelihoods of internally displaced patients on HD. When dialysis sessions became fewer or shorter, patients carried extra fluid, potassium, and uremic toxins to the next treatment; this is why symptoms and complications rose in our cohort. The risk is higher right after longer gaps between treatments (the first session after a 2-day break is known to carry more deaths and cardiac events).^[8]^ Extensive network data also show a dose–response: each missed or shortened session in a month is associated with increased hospitalization and mortality.^[9]^ Our pattern matches what has been reported during health system shocks and wars that disrupt dialysis schedules.^[5,6,10]^

Why were sessions missed? Dialysis depends on intact infrastructure (safe roads, electricity, clean water, supplies, and available staff). Conflicts damage or block these enablers, shut units, break supply chains, and make travel risky. Reviews of kidney care in crises describe these barriers and document that prolonged conflicts lead to sustained deviations from dialysis standards, resulting in worse outcomes.^[11]^ Field assessments from Syria show practical fixes that matter for displaced patients, including coordinated transport, temporary rationing with clear criteria, shared lists across units, and basic patient education for emergencies.^[12]^ Our survey echoes this: over 70% cited transport and money as the main reasons for missed dialysis, and more than 85% reported at least one missed session during active fighting.^[7,13]^ Recent preparedness guidance on kidney health (although focused on pediatrics) identifies similar priorities for protracted disruptions, emphasizing secure transport pathways, protection of water treatment and dialysate supply chains, and maintaining clinical communication when specialist coverage is limited.^[14]^

Are fewer sessions ever acceptable as a temporary bridge? Only if planned and monitored. Guidance allows less-than-thrice-weekly HD only when patients have meaningful residual kidney function and stable volume/electrolytes, with close checks and a plan to step up frequency,^[15]^ and with incremental HD frameworks that start lower and escalate as function declines.^[16,17]^ In our cohort, the change in frequency was an unplanned loss of access, not an incremental program, consistent with the excess complications we observed.^[8,9]^

Dialysis is time- and travel-dependent; when income collapses and transport is unsafe or unaffordable, adherence drops further, driving a loop of more missed sessions and worse health. This dynamic is well described in conflict settings, including Sudan and neighboring crises, where out-of-pocket costs and facility closures limit access even when distances seem manageable,^[7,18]^ and in crisis reviews calling for transport solutions, evacuation plans, stock buffers, and patient emergency kits.^[11]^

How our results fit broader evidence: Reports from Ukraine, Syria, and Yemen describe shortened or reduced treatment frequency and rising morbidity during periods of insecurity, findings that closely mirror our observations.^[5,6,10]^ A Syrian multi-center assessment documented unit closures, staffing gaps, and ad hoc workarounds, again matching the mechanisms observed in Port Sudan.^[12]^ Crisis reviews further note that chronic conflicts produce long-lasting damage to kidney care, in contrast to one-off natural disasters.^[11]^

These observations have practical implications. Care can be stabilized by secured transport or transport vouchers, a cross-center patient registry for displaced patients, small stock buffers for dialyzers/consumables, brief low-literacy education on actions after missed sessions, and (only if unavoidable) temporary twice-weekly HD under incremental-HD safety criteria with rapid return to thrice-weekly.^[16,17,19]^ These steps align with conflict-setting guidance^[19]^ and our earlier policy messages for Sudan,^[1,20]^ and they are consistent with preparedness recommendations to protect dialysis access and essential supplies during sustained disruptions.^[14]^

### 4.1. Limitations

Although a single-center study, Port Sudan became a major destination for displaced people during the conflict, so our sample draws from many regions. Still, the cross-sectional design supports associations rather than causation, a limitation shared with other crisis surveys.^[19]^ We did not capture delivered dose (Kt/V), potassium trajectories, or adjudicated clinical events. Future prospective, multi-center work with visit-level data (missed hours, delivered Kt/V, weight, and potassium trends) and outcomes will better quantify the cost of each missed session.

## 5. Conclusions

The 2023 Sudan conflict sharply worsened health and economic conditions for dialysis-dependent internally displaced persons in Port Sudan. Reduced frequency and shortened sessions (combined with unsafe or unaffordable travel) led to more fluid overload, electrolyte imbalance, and hospital use. This pattern is expected, given the minimum time needed for safe HD and the known link between missed/shortened treatments and worse outcomes.^[8,9,15]^ Immediate action (secured transport, financial support, reliable supplies, and pragmatic crisis protocols) can stabilize care. At the same time, conflict persists,^[11]^ and preparedness guidance offers concrete steps to safeguard dialysis continuity in protracted emergencies.^[14]^

## Author contributions

**Conceptualization:** Malaz Taha, Gaffar Hussein, Shahd Jaweesh, Faris Abdon.

**Data curation:** Osama Abbadi, Faris Abdon.

**Formal analysis:** Shahd Jaweesh, Osama Abbadi.

**Investigation:** Gaffar Hussein, Shahd Jaweesh.

**Methodology:** Malaz Taha, Gaffar Hussein.

**Supervision:** Osama Abbadi.

**Visualization:** Malaz Taha, Shahd Jaweesh.

**Writing – original draft:** Malaz Taha, Gaffar Hussein, Shahd Jaweesh, Osama Abbadi, Faris Abdon.

**Writing – review & editing:** Osama Abbadi, Faris Abdon.
